# RNA Processing and mRNA Surveillance in Monogenic Diabetes

**DOI:** 10.4137/grsb.s782

**Published:** 2008-05-21

**Authors:** Jonathan M. Locke, Lorna W. Harries

**Affiliations:** Institute of Biomedical and Clinical Sciences, Peninsula Medical School, Exeter, U.K

**Keywords:** splicing, isoforms, nonsense-mediated decay, MODY

## Abstract

In the eukaryotic cell a number of molecular mechanisms exist to regulate the nature and quantity of transcripts intended for translation. For monogenic diabetes an understanding of these processes is aiding scientists and clinicians in studying and managing this disease. Knowledge of RNA processing and mRNA surveillance pathways is helping to explain disease mechanisms, form genotype-phenotype relationships, and identifying new regions within genes to screen for mutations. Furthermore, recent insights into the regulatory role of micro RNAs (miRNAs) and RNA editing in the pancreas suggests that these mechanisms may also be important in the progression to the diabetic state.

## Introduction

The classical view of mRNA as a ‘passive’ messenger that simply transfers information from the form of DNA to protein has been challenged in recent years. An appreciation of the influence of mRNA stability and RNA processing in determining the nature and level of gene expression is growing, with their roles in an increasing number of diseases being elucidated. The aim of this article is to review the impact that an understanding of RNA processing and mRNA surveillance has had on monogenic diabetes and suggest where future developments may occur in this field.

### RNA processing

Following transcription, a pre-mRNA molecule is capped, spliced and polyadenylated. Whilst the addition of a cap structure to the 5′ end seems to occur on most mRNA molecules generated by RNA Polymerase II (RNAPII), whether normal or aberrant, the processes of splicing and polyadenylation are more tightly regulated and occur at variable positions along the pre-mRNA. These mechanisms thus have an important role in determining the nature of the transcript, and consequently the protein or proteins that may be produced.

### Splicing

Most mammalian genes consist of long, non-coding sequences (introns) that are separated by short, coding sequences (exons). In order to generate a functional message from the DNA template the pre-mRNA molecule composed of introns and exons must be spliced to generate the mature mRNA molecule consisting only of exons. This ‘cut and stick’ process is achieved by the coordinated action of five small nuclear RNAs (snRNAs) and more than 60 polypeptides in a macromolecular riboprotein complex called the spliceosome.

**Figure 1:**
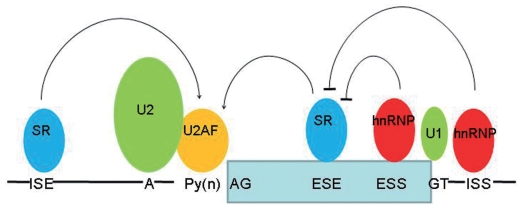
Splicing control elements Recognition of an exon is guided weakly by four cis-elements; the splice donor (GT), splice acceptor (AG), polypyrimidine tract (Py(n)) and branch Point (A) sequences. These are bound by the U1 and U2 small nuclear ribonucleproteins (snRNps) and the U2 auxillary factor protein (U2AF). Splicing regulatory (SR) proteins and heterogeneous nuclear ribonucleoproteins (hnRNPs) bind to enhancer (ESE/ISE) or silencer (ESS/ISS) elements and enhance or negate the recruitment of the U2AF component of the spliceosome to the polypyrimidine tract.

The recognition of an exon is guided by four weakly conserved intronic *cis*-elements: the 5′ splice site, 3′ splice site, the polypyrimidine tract and the branch site. These sequences form, by RNA:RNA base pairing, bonds with U1 and U2 small nuclear ribonucleoproteins (snRNPs) that form part of the spliceosome. Whilst necessary these *cis* elements are insufficient for accurate splicing; additional regulatory sequences within or near an exon act to enhance or silence exon recognition and consequently its presence/absence in the spliced transcript (Fig. 1). Enhancer sequences, termed exonic splicing enhancers (ESEs) or intronic splicing enhancers (ISEs), act as binding sites for specific serine/arginine-rich (SR) proteins. These proteins may aid in the recruitment of the U2AF component of the spliceosome complex or alternatively antagonise the action of nearby silencer elements. Silencer sequences, called exonic splicing silencers (ESSs) or intronic splicing silencers (ISSs), may be bound by members of the heterogeneous nuclear ribonucleoproteins (hnRNP) family. Several models are proposed to explain how these proteins inhibit exon definition. These include direct competition, where an ESE and ESS sequence overlap resulting in mutually exclusive protein binding, or nucleation whereby binding of one hnRNP causes subsequent polymerisation and occlusion of SR proteins from their target sites.

Alternative splicing allows for the production of multiple functionally distinct proteins (isoforms) from a single gene. It is estimated that approximately 74% of mammalian genes are alternatively spliced in at least one exon (Johnson et al. 2003). Variant transcripts might arise as a result of exon skipping, use of alternative 5′ or 3′ splice sites, or retention of an intron. Many alternative exons are designated by weak splicing signals; poorly defined polypyrimidine tracts are particularly common and have a greater requirement for SR proteins to recruit the U2AF complex. Regulation of alternative splicing is thus strongly dependent upon the balance of active-to-inactive SR proteins and hnRNPs (Stamm, 2007). Mainly regulated by phosphorylation, the activities of these proteins have been shown to differ in a tissue- and stage-specific manner (Stamm et al. 2005) and be dependent on extracellular signalling cues such as receptor-binding ligands (Wang et al. 1991); temperature (Poly, 1997) and pH (Borsi et al. 1995).

### Polyadenylation

Once RNAPII encounters a highly conserved polyadenylation signal (typically, but not exclusively, AAUAAA) the multi-protein complexes, Cleavage and Polyadenylation Specificity Factor (CPSF) and Cleavage Stimulation Factor (CstF), disembark from RNAPII and transfer to the pre-mRNA. CPSF binds to the polyadenylation signal sequence and CstF to the GU-rich sequence 20–60 base pairs 3′ to the polyadenylation signal. These two complexes then promote cleavage of the transcript approximately 35 nucleotides downstream of the polyadenylation signal. Upon cleavage, Poly (A) Polymerase (PAP) starts adding the polyadenosine tail of 100–250 base pairs and Poly (A) Binding Protein 1 (PABPN1) subsequently binds to this tail, helping to target the mRNA for nuclear export.

Alternate splicing events can result in mRNAs which use different polyadenylation signals and thus have distinct 3′ UTRs. These untranslated regions can contain a number of regulatory elements that affect mRNA turnover, such as AU-rich elements responsible for transcript stability, and miRNA target sequences involved in the regulation of mRNA translation.

### mRNA surveillance

Given that errors do arise in transcripts, it would be tempting to speculate that cells could be filled with defective mRNAs, which have the potential to be translated into aberrant proteins (Fasken and Corbett, 2005). However cells avoid the accumulation of many defective mRNAs by using a variety of quality control mechanisms, such as Nonsense-Mediated Decay (NMD) and Nonstop Decay (NSD).

**Figure 2:**
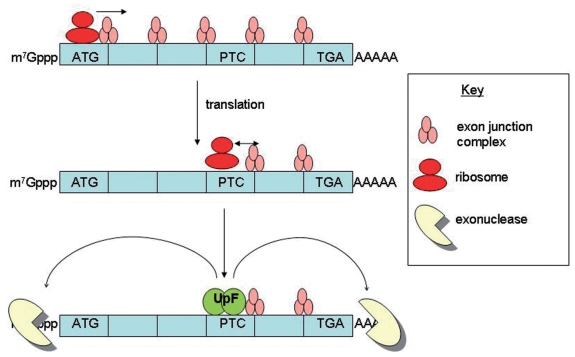
Nonsense-mediated decay model As a ribosome navigates along a transcript it is believed to remodel or displace the exon junction complexes. If, upon translating a premature termination codon, any exon junction complexes are left 3′ to this mark UPF proteins bind to the transcript. This serves to target the transcript for degradation by exonucleases.

The translation-dependent pathway, NMD, selectively degrades transcripts bearing a premature termination codon (PTC). About one-third of genetic and acquired diseases are believed to be the result of a PTC (Kuzmiak and Maquat, 2006). It is thought that NMD occurs when not all exon-junction complexes (EJCs), which are deposited on exon-exon borders during splicing, are removed by a pioneer round of translation in the nucleus. When navigating along a transcript without a PTC, the ribosome is believed to displace or remodel all EJCs. However if a transcript has a PTC, none of the EJCs 3′ to this mark are removed, and this serves as a signal for binding of the Up-frameshift (UPF) proteins to the transcript. These proteins mark the transcript for degradation through two main pathways; the 5′-3′ Xrn1 and 3′-5′ exosome-dependent pathways (Fig. 2) (Baker and Parker, 2004).

Recognition of a nonsense codon by the NMD pathway may also modulate the splicing machinery to eliminate production of transcripts bearing the PTC. In nonsense-associated altered splicing (NAS), the splicing of the pre-mRNA is modified so that the exon carrying the nonsense mutation is not included in the mature transcript. This may confer a selective advantage by allowing a protein of residual function to be made, which has been documented to moderate Duchenne muscular dystrophy (DMD) (Disset et al. 2006). Alternatively the splice variant may have deleterious consequences by acting in a dominant-negative fashion. Most frequently however NAS is likely to result in a change in the reading frame which in turn triggers NMD anyway. Several mechanisms are proposed to explain NAS, one suggesting the existence of a nuclear mRNA surveillance system distinct from the NMD machinery that verifies the integrity of an ORF. This, when necessary, directs the splicing machinery to skip the offending exon (Cartegni et al. 2002).

In contrast to NMD the nonstop decay (NSD) pathway recognises mRNAs that *lack* a termination codon resulting in abnormally extended transcripts. Whilst it is known that the mutant transcript is recognised and degraded by the Ski complex and cytoplasmic exosome (Fasken and Corbett, 2005), this pathway is not well characterised in humans, partly due to the rarity of mutations that result in ribosomal read-through of termination codons.

### Monogenic diabetes

As Type 2 diabetes reaches epidemic proportions it is worth restating that it is a diagnosis of exclusion, and that around 1%–2% of cases are likely to have a monogenic form of diabetes (Gloyn and Ellard, 2006). Recent progress in defining genes causing two forms of monogenic diabetes—maturity onset diabetes of the young (MODY) and neonatal diabetes (NDM)—are having profound implications on patient care and also providing fascinating insights into the aetiology of the more common Type 2 diabetes.

Maturity-onset diabetes of the young (MODY) is an autosomal dominant form of inherited diabetes characterised by beta-cell dysfunction. Patients presenting with the disease are usually slim, diagnosed at an early age (typically, but not exclusively <25 years) and not insulin-dependent (although insulin treatment may be required in the later stages of the disease). In ~89% of MODY cases a mutation in one of seven genes (HNF1A, HNF4A, HNF1B, GCK, NEUROD1, IPF1, and CEL) has been found (Frayling et al. 2001).

Neonatal diabetes is now defined as hyperglycaemia presenting within the first 6 months of life (Massa et al. 2005). Around 50% of these cases will have transient neonatal diabetes (TNDM) where diabetes resolves within this time frame (Gloyn and Ellard, 2006). TNDM is most commonly associated with abnormalities of an imprinted locus on chromosome 6q24 (Temple et al. 2000). The remaining half of cases will not resolve and are termed to have permanent neonatal diabetes (PNDM) where treatment is required for life. Approximately 41% of PNDM cases are caused by heterozygous activating mutations in either *KCNJ11* or *ABCC8*, the genes encoding the K_ATP_ channel subunits, Kir6.2 and SUR1 (Edghill et al. 2008). The K_ATP_ channel plays a key role in insulin secretion and functional characterisation of activating mutations in the Kir6.2 and SUR1 subunits have shown that mutant channels have a reduced sensitivity to ATP. This results in a failure of the K_ATP_ channel to close in response to ATP, and ultimately, the pancreatic beta cell to secrete insulin effectively. It has been recently identified that ~20% of PNDM cases are caused by mutations in the INS gene (Stoy et al. 2007).

## The Role of RNA Processing in Monogenic Diabetes

### Isoforms of HNF1A and HNF4A have an ability to moderate MODY phenotype

The *HNF1A*, *HNF4A* and *HNF1B* genes encode for multiple isoforms which are generated by a combination of alternate splicing, polyadenylation and promoter-usage events. The *HNF1A* gene codes for three isoforms that are identical in their DNA binding and dimerisation properties but have different transactivation potentials. The expression of these isoforms has been shown to differ in a temporal and spatial manner (Harries et al. 2006). Whilst *HNF1A(A)* is believed to be the most common isoform in most tissues (Bach and Yaniv, 1993), *HNF1A(B)* is predominant in both total adult pancreas and isolated islet cells. Levels of *HNF1A(C)* are also higher in these adult tissues (Harries et al. 2006).

The regulated expression of these isoforms suggested a functional role, particularly in tissues with high expression (i.e. the pancreas). Harries et al. hypothesised that mutations that affect only certain HNF1A isoforms might lead to different influences on beta-cell dysfunction and subsequently diabetic phenotype. In a cohort of 564 HNF1A MODY patients with 123 different mutations age of diagnosis was related to mutation position and isoform structure. We found that patients with a missense mutation in the first six exons, affecting all isoforms, presented with diabetes an average of 12 years before patients with a missense mutation in exons 8–10, which would only affect HNF1A(A) (Harries et al. 2006). This provided further proof for a functional role of isoforms HNF1A(B) and (C) in the pancreas.

A similar situation has been subsequently shown with regards to *HNF4A* mutation position and age of diagnosis (Harries et al. 2008). Patients with a mutation in exon 9 or 10 of *HNF4A* (that does not affect isoforms HNF4A(3), (6) or (9)) present at a median age of 40. This is 16 years later than the median age of onset for patients with a mutation in exons 2–8, that affects all isoforms. Perhaps of more interest to the clinical setting was the analysis relating mutation position to age-related penetrance. Using a Kaplan-Meier analysis it was shown that by 55 years of age 53% of patients with a mutation in exons 9 and 10 had not gone on to develop diabetes compared with just 9% of patients with a mutation in exons 2–8. These findings have important consequences for the management and monitoring of families with less penetrant mutations. This study also challenges the longstanding definition of MODY as a disease that will typically present within the first 25 years of life.

### Discovering new areas to screen for mutations

When screening a gene for disease-causing mutations intronic variants outside the conserved splice donor and acceptor sites are often overlooked as being potentially pathogenic. In genetic testing for MODY there seems to be an awareness for the potential of mutations to affect *cis* elements regulating constitutive splicing, outside the conserved 5′ and 3′ splice sites. For example the *HNF1A* IVS7–6G>A mutation was shown to result in the generation of an alternative splice acceptor site and a frameshift that resulted in a PTC (Bulman et al. 2002).

An increasing appreciation for the importance of alternate RNA processing in the *HNF* genes also opens up new gene regions in which to screen for mutations causing MODY. The finding that the predominant HNF4A isoforms in the adult pancreas are transcribed from an alternative promoter (P2) to that in the adult liver (P1) highlighted a new area to screen. Also involving an alternative splicing event, with the alternate first exon (exon 1D) spliced to exon 2, mutations in the P2 promoter and exon 1D have subsequently been shown to cause MODY (Thomas et al. 2001; Ellard and Colclough, 2006). No mutations in the P1 promoter and exon 1A, coding in isoforms predominantly expressed in the liver, have been found to cause MODY. This stresses the need to determine expression profiles of isoforms when embarking on genetic testing.

Both HNF1A(B) and HNF1A(C) contain novel amino acids derived from intronic sequences and it seems plausible that mutations in these regions, not currently screened by sequencing, could cause MODY. Despite having truncated transactivation domains these shorter isoforms have been shown to possess higher transactivation activities than the full length transcript (HNF1A(A)) (Bach and Yaniv, 1993). The two shorter isoforms also provide approximately 75% of *HNF1A* expression in the adult pancreas. Similarly the translated intronic sequences in certain HNF1B and HNF4A isoforms might also be investigated for possible mutations. The report by Harries et al. suggests an important role for HNF4A(3) in the pancreas and so mutations causing MODY may be found in IVS8, part of which is coding in this isoform (Harries et al. 2008).

### An understanding of splicing helps to explain the molecular mechanisms causing the disease

*HNF1B* gene mutations affect development of the kidneys and pancreas, resulting in renal developmental abnormalities, reduced birth weight and diabetes in later life (Bingham and Hattersley, 2004; Edghill et al. 2006). The most common renal manifestation is renal cysts which are frequently diagnosed on antenatal ultrasound. This syndrome is known as renal cysts and diabetes (RCAD), but can also include female genital tract malformations, gout and liver disease (Bingham and Hattersley, 2004). The finding that the HNF1B gene encodes an isoform that acts as a transcriptional repressor (HNF1B(C)) may explain why most mutations in this gene are in exons 1–4. HNF1B(C) is transcribed from only these four exons and one would postulate that a mutation in one of these exons would inactivate it. A missense mutation elsewhere in the gene would not affect the repressor and could lead to loss of HNF1B activity not only from the mutant allele but also sequestration of product from the wild-type allele in inactive dimers with HNF1B(C). Whilst missense mutations in exons 5–10 have not been shown to cause RCAD insertion/deletion mutations have been reported. These mutations were shown to be highly susceptible to NMD (Harries et al. 2005) and would result in some reduction of HNF1B(C) levels. This idea that slight overexpression of full-length HNF1B causes a decrease in insulin secretion is supported by studies in rat INS-1 cells. Conditional expression of HNF1B led to loss of the insulin secretory response whilst expression of a dominant-negative form did not (Welters et al. 2006).

Determining the functional effect of mutations in GCK has centred around their effect on protein kinetics and stability. Indeed the majority of mutations in GCK seem to exert their deleterious effect through reducing protein stability or reducing the enzymes affinity for glucose. However certain mutations such as A53S, H137R and R275C seem to show normal protein characteristics (Matschinsky, 2002). Several theories abound as to how these mutations cause MODY. One such reason might be their ability to cause aberrant splicing through disruption of ESE sequences. Approximately 15% of ‘missense’ mutations are thought to exert their effect through altered splicing rather than protein function (Nissim-Rafinia and Kerem, 2005).

## mRNA Surveillance and Monogenic Diabetes

### An understanding of NMD helps to explain the molecular mechanisms causing the disease

As MODY is an autosomal dominant disease, where only one allele is mutated, it had been proposed that the clinical phenotype be caused either by haploinsufficiency or by a dominant-negative effect of the mutated protein upon the wild-type protein. Whilst initial *in vitro* characterisation of the common P291fsinsC mutation in *HNF1A* suggested a dominant-negative mechanism (Yamagata et al. 1998), it has been subsequently shown that MODY is most likely a disease of haploinsufficiency (Harries et al. 2004). Clues for this came from the similar phenotypes of patients with HNF1A MODY due to loss-of-function mutations (e.g. promoter or dimerisation domain mutations) and patients with the initially presumed dominant-negative P291fsinsC mutation. Furthermore the demonstration that NMD results in the destruction of several transcripts bearing PTCs, and that whole *HNF1A* gene deletions have been documented to cause MODY, proves it is a haploinsufficiency syndrome. RCAD caused by HNF1B mutations is also likely to be a disease of haploinsufficiency as whole gene deletions of *HNF1B* have been documented (Edghill et al. 2007), and a number of mutations in *HNF4A* have also been shown to be loss-of-function (Yang et al. 2000).

One must be careful though when stating whether a disease is one characterised solely by haploinsufficiency or dominant-negative mechanisms. The numerous reports documenting differences in the degree of NMD shows that it is not an all-or-nothing process (Wang et al. 2002; Inacio et al. 2004; Harries et al. 2005). A percentage of PTC-containing transcripts will remain, with this number often related to the position of the PTC within the gene. As a result different mutations can vary quite markedly in their susceptibility to NMD. In addition to NMD, translation reinitiation events, and possible NAS, may also increase the number of potentially dominant-negative proteins produced. In one diabetic family with an *IPF-1* frame shift mutation a novel isoprotein has been shown to be generated as a result of translation reinitiation. It was subsequently shown to exert a dominant-negative effect on the activation of transcription by the wild-type protein (Stoffers et al. 1998). Despite apparently being a disease of haploinsufficiency the significant differences in NMD susceptibility seen between PTC-causing mutations in *HNF1B* suggests dominant-negative proteins may be generated. Indeed examination of the 5′ end of the gene shows the presence of two potential reinitiation codons each displaying some conservation of the surrounding sequence necessary for translation (Harries et al. 2005). Generation of truncated isoforms has the potential to moderate phenotype and may partly explain the phenotypic heterogeneity associated with *HNF1B* mutation carriers.

In PNDM an understanding of the role of NMD has helped explain how a mildly activating *ABCC8* mutation can result in diabetes when a loss-of-function mutation is inherited on the other allele. In a patient who had inherited a frameshift mutation on one allele and a mildly activating mutation on the other, NMD was shown to result in significant degradation of the transcript from the frameshift allele. As a result it was proposed that the vast majority of pancreatic K_ATP_ channels would be homomeric for the missense mutation. This was the first reported disease phenotype to be caused by compound heterozygosity for both gain-of-function and loss-of-function mutations (Ellard et al. 2007).

## Future Considerations

### MicroRNAs

MicroRNAs (miRNAs) are short 21–22 nucleotide RNA molecules that bind in a sequence-specific manner to the 3′ untranslated region (UTR) of target mRNAs. Believed to be dependent on the miRNA-mRNA sequence match, this association either impairs translation initiation or causes mRNA degradation. miRNAs have been shown to regulate genes involved in a myriad of cell processes such as apoptosis, proliferation, differentiation and neuronal cell fate (Gauthier and Wollheim, 2006). They have also been associated with cancer and neurological disorders (Jin et al. 2004; Lu et al. 2005).

In a study by Poy et al. the pancreatic islet-specific miRNA, *miR-375*, was shown to regulate insulin exocytosis, pointing to a possible involvement of microRNAs in diabetes (Poy et al. 2004). Using a siRNA homologous to *miR-375* it was shown that insulin secretion was significantly impaired, to a level similar to that of siRNA-mediated knockdown of *GCK*. This was one of the first accounts of a single miRNA having a measurable phenotypic effect. It had previously been thought that miRNAs had overlapping functions each only acting to ‘fine-tune’ gene expression (Stark et al. 2005). Subsequently *miR-9* and *miR-124a* have been implicated in regulating insulin secretion and intracellular signalling pathways in beta cells (Plaisance et al. 2006; Baroukh et al. 2007). *miR-375* has also been shown to play a crucial role in maintaining normal islet cell architecture during embryonic development (Kloosterman et al. 2007). A mouse conditional knockout of the miRNA-processing enzyme, Dicer1, specifically in early pancreas development leads to significant beta cell loss (Lynn et al. 2007). Given the significant role of several microRNAs in the pancreas it may be that mutations in miRNA genes could cause a monogenic form of diabetes. Indeed it may be worth revisiting MODY linkage scans, where no causative protein-coding gene was found, for possible miRNA involvement.

If mutations in miRNAs or their target sites do not directly cause diabetes information regarding their target genes may however be of use. Combining data from expression profiles of miRNAs and their target mRNAs may help in constructing a more considered list of candidates to screen as possible disease-causing genes. Recent technological advances mean that spatial and temporal profiling of all known human miRNAs can be done easily and quickly. This can be combined with our knowledge that specific miRNAs target genes involved in the same complex cell processes, e.g. insulin exocytosis. For example, if a miRNA was shown to target all members of the *HNF* family causing MODY and another gene also expressed largely in beta cells then this gene might become a strong candidate gene. Several of the *HNF* isoforms do possess long (>1000 bp) 3′ UTRs which contain multiple predicted miRNA binding sites. *HNF1B* is a particularly likely candidate for miRNA regulation as whilst its transcripts are abundantly present in adult pancreatic islets there seems to be limited expression of this protein in mature beta cells (Maestro et al. 2003). miRNA-mediated regulation of *HNF1B* is likely to be a sensible way of tightly regulating HNF1B protein levels. Too much HNF1B is associated with apoptosis and a decrease in insulin secretion (Welters et al. 2006) whilst lack of expression is known to cause hyper proliferation and is associated with clear-cell and ovarian carcinomas (Rebouissou et al. 2005; Terasawa et al. 2006).

### RNA editing

RNA editing through the conversion of adenosine (A) to inosine (I) within pre-mRNAs is a genetic recoding process found in a variety of species including humans. A-to-I RNA editing is catalysed by a family of ubiquitously expressed enzymes, adenosine deaminases acting on RNA (ADARs). Nucleotide changes can arise in coding sequences and also alter pre-mRNA splicing and mRNA stability.

RNA editing was initially shown to play an essential role in the brain, regulating the activities of several neurotransmitter receptors and ion channels. The high similarity in cellular development, and expression of similar genes, suggested a role for A-to-I RNA editing by ADARs in the endocrine system. Gan et al. have shown that ADAR2 is significantly expressed in mouse islets and furthermore that its activity is regulated by the energy metabolism status of the cell (Gan et al. 2006). They report that one of its target genes is the glutamate receptor and that editing is increased in diet-induced obesity mice. Whether the nucleotide changes resulted in any alteration in glutamate signalling and consequently insulin secretion was not however determined. Like microRNAs it has been suggested that RNA editing may control several components of the insulin release machinery (Gan et al. 2006). Combined computer-based and experimental advances are improving our ability to identify RNA editing substrates; recently C-to-U RNA editing sites have been predicted *in silico* with a stated 84% accuracy using solely nucleotide sequence features (Du et al. 2007).

### Therapeutic intervention at the RNA level

Gene silencing strategies have the potential to treat a variety of diseases by addressing targets that had previously been thought of as ‘undruggable’. The relative ease with which mRNA can be accessed has resulted in most therapeutic efforts being concentrated at the RNA level (Gewirtz, 2007). The principle surrounding these ‘antisense’ technologies is essentially the same; involving the delivery of a strand of DNA or RNA into a cell that is reverse complementary to the mRNA encoding the protein that one would like to destroy.

Whilst neonatal diabetes caused by mutations in the *KCNJ11* and *ABCC8* genes seems to be successfully controlled by high dose sulphonylurea treatment (Pearson et al. 2006; Rafiq et al. 2007) there is a need for more efficacious treatment caused by mutations in other genes. For example, most INS mutations are believed to cause diabetes due to a loss of beta-cell function and mass, that is the result of the mutant dominant-negative acting INS allele (Stoy et al. 2007). Targeted degradation of mutant INS alleles might thus be advantageous. Recent research indicates that small interfering RNAs (siRNAs) could be developed to discriminate between wild-type and mutant alleles that differ by just a single nucleotide (Schwarz et al. 2006). A study in mice has described the successful introduction, by intravenous tail vein injection, of an siRNA targeting *Ins2* into islet cells which resulted in a 33% reduction in *Ins2* transcript levels (Bradley et al. 2005).

Being dominantly inherited diseases classically defined as haploinsufficiency syndromes, the various subtypes of MODY are perhaps not the most attractive candidates for antisense therapies that are typically aimed at *decreasing* transcript levels. Despite this there are perhaps a couple of avenues where therapies acting at the RNA level may be of use.

Firstly, frameshift-causing mutations in exons 5–10 of *HNF1B* could be targeted by antisense-mediated exon skipping. Frameshift mutations in these exons likely cause diabetes by producing a PTC which activates NMD and degradation of the entire mutant allele. The absence of missense mutations causing MODY in exons 5–10 of *HNF1B* suggests that not all of these exons are necessary for correct HNF1B function. By designing antisense oligonucleotides against the conserved splice sites of these exons it may be possible to skip the non-essential exon containing the nonsense mutation whilst maintaining the correct reading frame. Encouraging results for this exon skipping technique have been reported in a Duchenne muscular dystrophy mouse model with a nonsense mutation in exon 23 of the dystrophin gene (Lu et al. 2005).

Secondly, a new molecular weight compound, called PTC124, is being developed that could be used to treat multiple diseases caused by nonsense mutations. PTC124 increases levels of transcript originating from a mutant allele harbouring a PTC by promoting ribosomal readthrough of the nonsense mutation, thus decreasing NMD. Inducing readthrough of premature, but not normal termination codons, this drug has been shown to rescue striated muscle function in mice modelling Duch-enne muscular dystrophy within 2–8 weeks of drug exposure (Welch et al. 2007).

## Concluding Remarks

The need to maintain strict control of HNF1A, 4A and 1B protein levels can be evidenced by the number of phenotypes associated with varied expression of these transcription factors. Variation in the P2 promoter of *HNF4A*, presumably leading to a decrease in HNF4A expression, has been associated with an increased risk of developing Type 2 diabetes (Weedon et al. 2004), whilst bi-allelic inactivation of *HNF1A* and *HNF1B* has been shown to cause a number of types of cancer (Bluteau et al. 2002; Terasawa et al. 2004; Rebouissou et al. 2005). As it has for monogenic diabetes, continued research in the area of RNA processing and mRNA surveillance may provide fascinating insights into the pathogenesis behind these diseases as well as other diabetes-related phenotypes such as renal disease and hyperinsulinaemic hypoglycaemia.
